# CACYBP Enhances Cytoplasmic Retention of P27^Kip1^ to Promote Hepatocellular Carcinoma Progression in the Absence of RNF41 Mediated Degradation

**DOI:** 10.7150/thno.36838

**Published:** 2019-10-22

**Authors:** Yi-Fan Lian, Yan-Lin Huang, Yao-Jun Zhang, Dong-Mei Chen, Jia-Liang Wang, Huan Wei, Yan-Hua Bi, Zhi-Wu Jiang, Peng Li, Min-Shan Chen, Yue-Hua Huang

**Affiliations:** 1 Guangdong Provincial Key Laboratory of Liver Disease Research, the Third Affiliated Hospital of Sun Yat-sen University, Guangzhou, China; 2 Department of Infectious Diseases, the Third Affiliated Hospital of Sun Yat-sen University, Guangzhou, China; 3 Department of Hepatobiliary Surgery, Sun Yat-sen University Cancer Center, Guangzhou, China; 4 Guangzhou Institutes of Biomedicine and Health, Chinese Academy of Sciences, Guangzhou, China

**Keywords:** Hepatocellular carcinoma, CACYBP/SIP, RNF41/Nrdp1, P27^Kip1^, Cell cycle

## Abstract

Calcyclin-binding protein (CACYBP) is a multi-ligand protein implicated in the progression of various human cancers. However, its function in hepatocellular carcinoma (HCC) remains unknown.

**Methods**: The expression of CACYBP and RNF41 (RING finger protein 41) in HCC cancer and adjacent non-tumor tissues was detected by immunohistochemistry. CCK-8 assays, colony formation assays, flow cytometry detection and xenograft models were used to evaluate the impact of CACYBP expression on HCC cell growth, apoptosis and cell cycle regulation. Immunoprecipitation and ubiquitination assays were performed to determine how RNF41 regulates CACYBP. The regulatory mechanism of RNF41-CACYBP signaling axis on P27^Kip1^ was investigated by western blotting and immunofluorescence.

**Results**: CACYBP was highly expressed and associated with poor prognosis in HCC. CACYBP expression was required for HCC cell growth* in vitro* and *in vivo*. Moreover, we identified RNF41 as a specific binding partner of CACYBP at exogenous and endogenous levels. RNF41 recruited CACYBP by its C-terminal substrate binding domain, subsequently ubiquitinating CACYBP and promoting its degradation in both proteasome- and lysosome-dependent pathways. In HCC tissues, RNF41 expression was reduced and conferred a negative correlation with CACYBP expression. Mechanistically, CACYBP overexpression stimulated the Ser10, Thr157 and Thr198 phosphorylation of P27^Kip1^ and its cytoplasmic retention, and RNF41 co-expression attenuated this phenomenon. CACYBP depletion led to decreased levels of cyclin D1, cyclin A2, CDK2 and CDK4, causing a typical cell cycle arrest at G1/S phase and increasing apoptosis in HCC cells. P27^Kip1^-S10D but not P27^Kip1^-S10A reconstitution rescued partially the cell cycle function and apoptotic feature after CACYBP depletion.

**Conclusion**: Our findings provide novel insights into the functional role and regulatory mechanism of CACYBP in HCC.

## Introduction

Hepatocellular carcinoma (HCC) is the most common malignant tumor in the liver, accounting for approximately 750,000 deaths worldwide annually 1. Despite progress in treatment options such as liver transplantation, surgery and adjuvant therapy, the five-year overall survival (OS) rate of HCC patients, especially at the intermediate and advanced stages, remains extremely low 2. A better understanding of the molecular mechanisms underlying liver carcinogenesis may allow the identification of novel molecular markers for HCC progression and development of new targeted therapies.

The term CACYBP comes from Calcyclin-binding protein (CacyBP) after the protein was identified as a binding partner of S100A6 in Ehrlich ascites tumor cells 3, and later was found as a Siah1 interacting protein in humans and termed SIP 4. For clarity, we use its official gene symbol CACYBP in humans to refer to this protein in this article. CACYBP participates in cellular processes such as proliferation, differentiation, cytoskeletal re-organization or protein ubiquitination through interacting with various protein partners 5. CACYBP consists of three structurally independent domains: a helical hairpin domain located at the N-terminus (residues 1-73), a central CS (CHORD and Sgt1) domain (residues 74-151) and a flexible C-terminal SGS (Sgt1-specific) domain (residues 152-229) 6. *Via* different binding sites, CACYBP interacts with four major groups of proteins 5: (1) E3 ligase-associated complexes, like Siah-1, Skp1 and TBL1; (2) S100 family proteins, like S100A6; (3) cytoskeletal proteins, like tubulin and actin; (4) other signaling molecules, like ERK1/2 and tau. Previous reports have also shown that CACABP is ubiquitously expressed in all kinds of tumor tissues 7 and contributes to the progression of a wide range of human malignancies including gastric cancer 8, pancreatic cancer 9, colon cancer 10, breast cancer 11, brain cancer 12 and renal cancer 13. To date, however, there is little evidence on the role and clinical significance of CACYBP expression in HCC.

RNF41 (RING finger protein 41) or Nrdp1 (neuregulin receptor degradation protein-1) is an E3 ubiquitin ligase mediating the ubiquitination and degradation of numerous protein targets in either proteasomal or lysosomal pathway. The reported downstream targets of RNF41 include BRUCE, an inhibitor of apoptosis 14; parkin, a signaling molecule involved in Parkinson's disease 15; RARα (retinoic acid receptor alpha), a receptor important for erythroid and myeloid differentiation 16; and the growth factor receptors ERBB3 and ERBB4, independent of their respective ligands 17, 18. RNF41 recruits E2 conjugating enzymes with its N-terminal RING finger domain (residues 18-57), followed by a coiled-coil domain essential for homotrimerization (residues 135-179), and interacts with the substrates *via* its C-terminal domain (residues 193-317) 17, 19. In addition to promoting degradation, ubiquitination by RNF41 also controls the sorting, processing and subcellular relocalization of the substrates, as demonstrated for LR (leptin receptor), LIFR (leukemia inhibitory factor receptor), IL-6R (interleukin-6 receptor) 20, USP8 21 and VPS52 22. RNF41 exerts differential ubiquitination functions towards Myd88 (poly-lys48) and TBK1 (poly-lys63) and regulates TLR-mediated production of type-I interferon 23. Loss of RNF41 has been reported in human cancers, such as breast cancer 24, prostate cancer 25, pancreatic cancer 26, glioma 27 and recently HCC 28. Though RNF41 regulates a broad range of physiological processes, its relationship with CACYBP and the underlying mechanism in HCC progression remain to be elucidated.

Here, we hypothesized that CACYBP is involved in unrestrained HCC growth, and examined the contribution of CACYBP activity to HCC progression. Furthermore, because CACYBP degradation is regulated by both proteasome and lysosome through the complex of CACYBP and RNF41, we determined the behavior of these regulatory mechanisms and the downstream signaling events mediated by RNF41-CACYBP axis. Finally, we evaluated the role of CACYBP as a prognostic marker and therapeutic target for human HCC.

## Results

### Increasing expression of CACYBP is associated with poor prognosis in HCC

**Figure 1:**
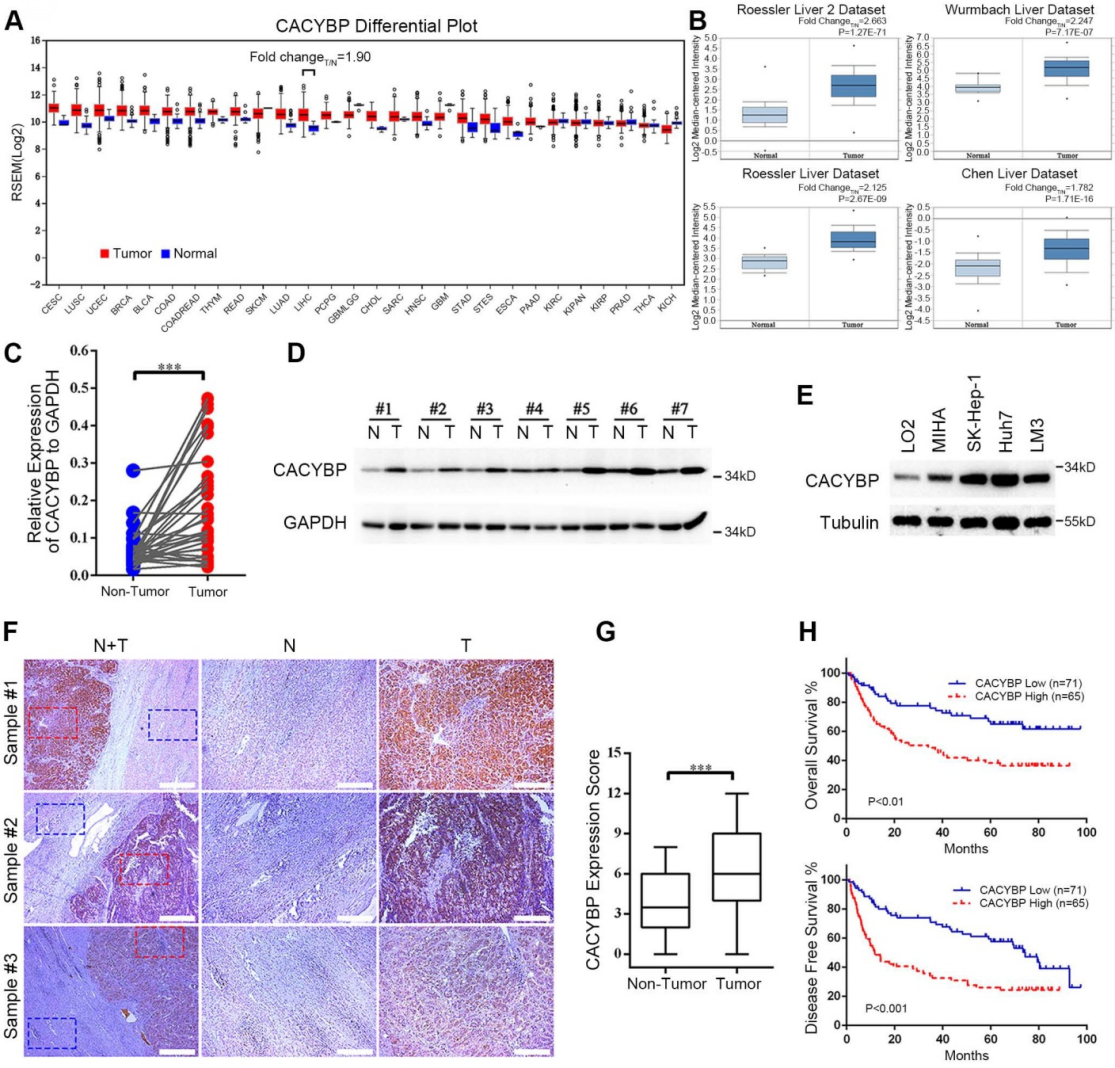
** Increasing expression of CACYBP is associated with poor prognosis in HCC.** (A) CACYBP mRNA levels in global human cancer tissues (red) and non-tumor tissues (blue) were analyzed using the TCGA database (http://firebrowse.org/). Noticeably, the fold change of tumor *vs* normal tissue in hepatocellular carcinoma was 1.90. CESC: Cervical squamous cell carcinoma and endocervical adenocarcinoma; LUSC: Lung squamous cell carcinoma; UCEC: Uterine corpus endometrial carcinoma; BRCA: Breast invasive carcinoma; BLCA: Bladder urothelial carcinoma; COAD: Colon adenocarcinoma; COADREAD: Colon and rectum adenocarcinoma; THYM: Thymoma; READ: Rectum adenocarcinoma; SKCM: Skin cutaneous melanoma; LUAD: Lung adenocarcinoma; LIHC: Liver hepatocellular carcinoma; PCPG: Pheochromocytoma and paraganglioma; GBMLGG: Glioblastoma multiforme and brain lower grade glioma (GBM + LGG); CHOL: Cholangiocarcinoma; SARC: Sarcoma; HNSC: Head and neck squamous cell carcinoma; GBM: Glioblastoma multiforme; STAD: Stomach adenocarcinoma; STES: Stomach and esophageal carcinoma; ESCA: Esophageal carcinoma; PAAD: Pancreatic adenocarcinoma; KIRC: Kidney renal clear cell carcinoma; KICH: Kidney chromophobe; KIRP: Kidney renal; KIPAN: Pan-kidney cohort (KICH + KIRC+ KIRP); PRAD: Prostate adenocarcinoma; THCA: Thyroid carcinoma. (B) CACYBP mRNA was elevated in HCC tissues compared with normal tissues in four datasets from ONCOMINE (https://www.oncomine.org/). The fold change and P value of the t-test for each dataset are shown. (C) CACYBP mRNA levels from 33 pairs of HCC tissues and matched adjacent non-tumor tissues were tested by qPCR. GAPDH was used as an internal control. (D) CACYBP protein levels from 7 pairs of HCC tissues and matched adjacent non-tumor tissues were tested by western blotting. (E) CACYBP expression in immortalized hepatic cell lines and HCC cell lines was analyzed by western blotting. (F) Representative images of CACYBP expression from three HCC tissues and their matched adjacent tissues by immunohistochemistry analysis. Scale bar for the left column: 400 μm; Scale bar for the middle and right columns: 100 μm. T: tumor; N: non-tumor. (G) Quantification of CACYBP expression scores in tumor tissues and non-tumor tissues from 52 HCC slices. (H) CACYBP expression was significantly associated with OS (upper panel) and DFS (lower panel) in the HCC cohort according to Kaplan-Meier analysis. CACYBP high: 71 samples; CACYBP low: 65 samples. ***P < 0.001.

To obtain a whole picture of CACYBP expression in human malignancy, we first searched the TCGA database and found that CACYBP mRNA expression was high in cancer tissues from most of the listed cancer types. Particularly in HCC, CACYBP expression was 1.9-fold higher in tumor compared to normal tissues (Figure 1A). We also looked up its expression in another open-access database, ONCOMINE. The results revealed that CACYBP expression was 2.663-fold, 2.247-fold, 2.125-fold, and 1.782-fold higher in HCC tumors from Roessler Liver 2 dataset, Wurmbach Liver dataset, Rossler Liver dataset and Chen Liver dataset, respectively, than in normal tissues (Figure 1B). Consistent with the above results, by examining paired fresh HCC tumor and adjacent non-tumor tissues, we confirmed that CACYBP mRNA and protein were also significantly upregulated in these tumor samples (Figure 1C and 1D). In addition, CACYBP was upregulated in HCC cell lines SK-Hep-1, Huh7 and LM3, compared to the immortalized hepatic epithelial cell lines LO2 and MIHA (Figure 1E).

We then asked whether CACYBP expression was associated with patient prognosis in HCC. We detected CACYBP expression in tissue slices resected from 136 cases of HCC tumors. CACYBP protein also showed an upregulated pattern in the tumor sections among these samples (Figure 1F and 1G). We grouped these patients into CACYBP high (n = 65) and low (n = 71) expression cohorts according to a cutoff value of the ROC (receiver operating characteristic) curve. Correlation analysis was used to examine the associations between clinicopathological features and CACYBP expression. The results revealed that high CACYBP expression was significantly correlated with elevated AFP level, elevated AST level, low ALB level, increased dead and recurrence events (Table 1). Cox hazard ratio analysis also confirmed that CACYBP expression was an independent factor significantly associated with HCC patient survival (Table S1). Moreover, Kaplan-Meier survival analysis indicated that higher CACYBP expression was significantly associated with shorter OS (P < 0.01, Figure 1H) and DFS (disease free survival, P < 0.001, Figure 1H) in our HCC patients. Overexpression of CACYBP also showed a significant correlation with reduced OS in HCC cohort from KMPlotter website (P < 0.01, Figure S1). Taken together, these results demonstrate that CACYBP expression not only is upregulated but also has a strong impact on the survival time of HCC patients.

### Knockdown of CACYBP expression impairs HCC cell growth* in vitro* and *in vivo*

**Figure 2:**
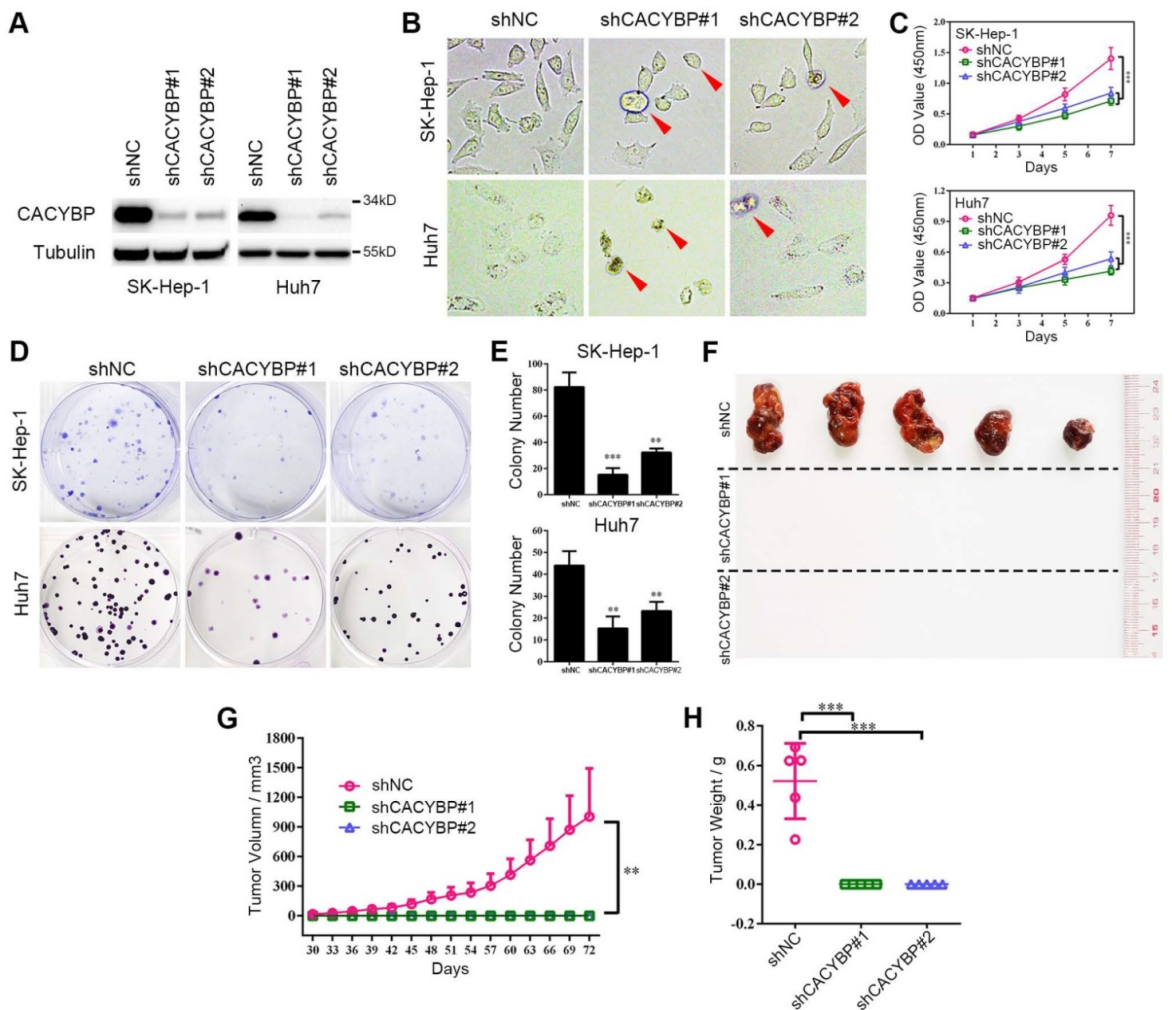
** Knockdown of CACYBP expression impairs HCC cell growth* in vitro* and *in vivo*.** (A) Western blotting analysis of the knockdown efficacy of shCACYBP#1 and shCACYBP#2 in SK-Hep-1 and Huh7 cells. (B) Representative images of control and CACYBP depletion cells under light field. Red arrows indicate the apoptotic-like cells. (C) Viability of SK-Hep-1 and Huh7 cells after CACYBP depletion was assessed by CCK-8 assay at indicated times. (D) Representative images of colony formation assays of SK-Hep-1 and Huh7 cells after CACYBP depletion. (E) Quantification of the colony numbers in (D). (F) All tumors isolated from NSI mice were shown. (G-H) Growth curves (G) and weights (H) of xenograft tumors from NSI mice injected with control and CACYBP-depleted SK-Hep-1 cells. Changes in tumor volumes measured on the indicated days are shown. **P < 0.01; ***P < 0.001.

To verify the function of CACYBP expression in HCC, we constructed stably knockdown HCC cell lines using two different CACYBP-targeting shRNAs. Knockdown efficiency was confirmed by western blotting, which showed that CACYBP expression was significantly depleted in SK-Hep-1 and Huh7 cells after shRNA treatment (Figure 2A). In these two cell lines, CACYBP depletion caused a rounded and shrinking cell shape, which showed an apoptotic-like morphological change, while the control cells were unaffected (Figure 2B). *In vitro* cell viability assay demonstrated that the proliferation of CACYBP-depleted cells was largely reduced (Figure 2C) and the colony number of CACYBP-depleted cells was 2- to 3-fold lower compared to the control cells (Figure 2D and 2E). We also constructed CACYBP-overexpressing SK-Hep-1 and LO2 cells after infection with the lentivirus encoding Flag-tagged CACYBP (Figure S2A). The results from the *in vitro* assays demonstrated that CACYBP overexpression had no significant effect on the proliferation and colony formation abilities in SK-Hep-1 cells, but promoted these growth abilities in LO2 cells (Figure S2B-D). LO2 is a non-cancerous hepatic cell line, although some reported that LO2 itself could form xenograft tumors in nude mice 29, 30. However, we observed no tumor formation even at month 2 after subcutaneously injecting either LO2 control or CACYBP-overexpressing cells into NSI mice (data not shown). The above results indicated that CACYBP overexpression enhanced cell growth ability in pre-cancerous hepatic cells, but would not lead to tumor transformation.

In addition, we evaluated the effect of CACYBP depletion *in vivo* by subcutaneously injecting SK-Hep-1 control, shCACYBP#1 or shCACYBP#2 cells into NSI mice. Noticeably, neither shCACYBP#1 nor shCACYBP#2 group formed palpable tumors while all 5 mice in the control group did at the injection sites (Figure 2F and Figure S3). Tumors in the control group appeared 30 days after injection and grew normally. However, even at day 72 after injection, no observable tumors could be measured in CACYBP knockdown groups (Figure 2G and 2H). Taken together, the above results demonstrate that CACYBP expression is required for HCC cell growth *in vitro* and *in vivo*.

### E3 ubiquitin ligase RNF41 co-immunoprecipitates with CACYBP

**Figure 3:**
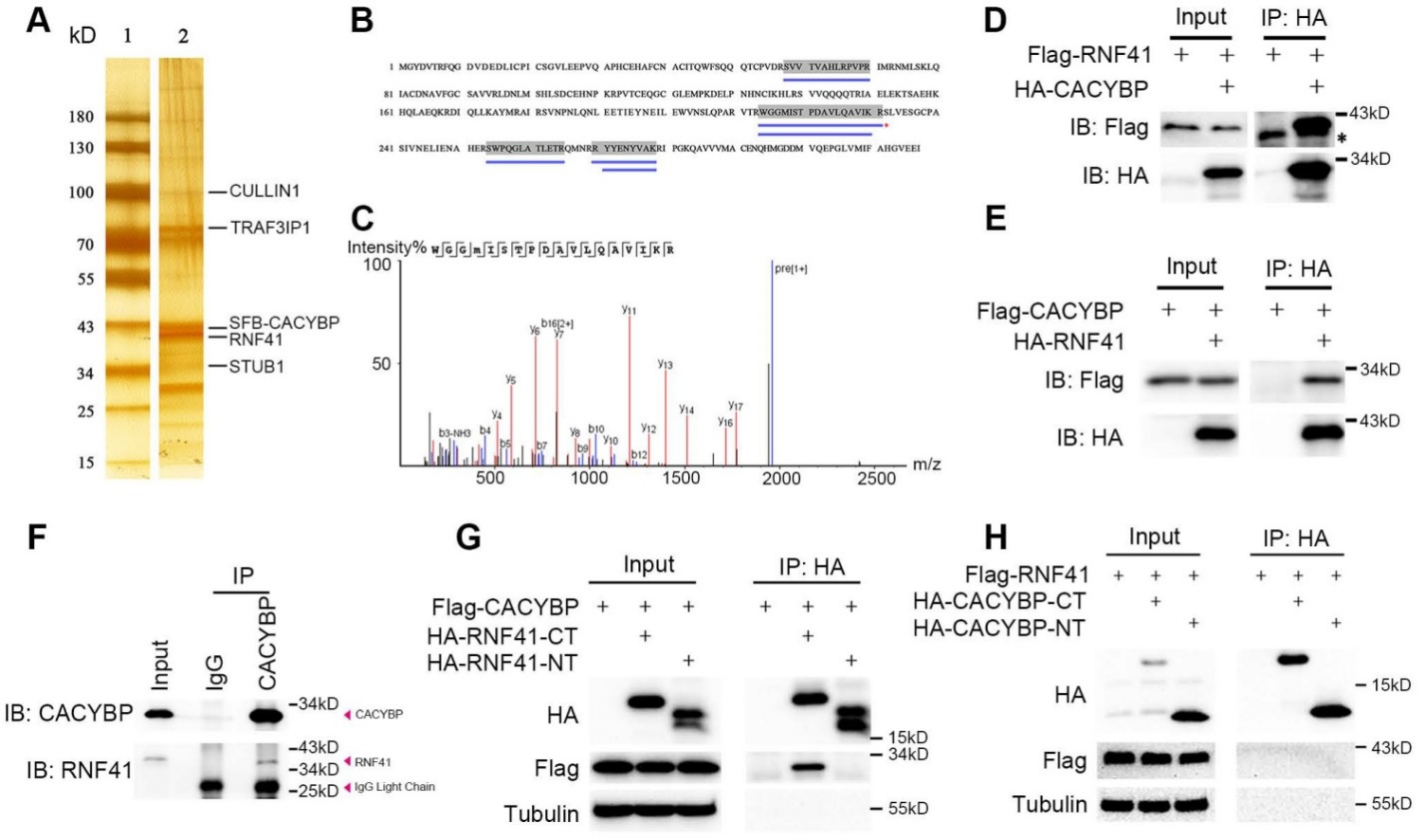
** E3 ubiquitin ligase RNF41 co-immunoprecipitates with CACYBP.** (A) Silver-stained SDS-PAGE gel of protein marker only (lane 1) and the immunoprecipitated complex after a two-step TAP from SFB-CACYBP expressing 293T cells (lane 2). Bands were excised from the gel, trypsin-digested, and analyzed by MALDI-TOF. Identified proteins are indicated by lines. (B) Protein coverage of RNF41 in the MS result. The blue lines under the amino acid sequence indicate the supporting peptides matching RNF41. (C) MS sequencing of the best unique peptide (indicated by a red asterisk in (B)) of RNF41 protein. (D-E) Immunoprecipitation and western blotting experiments were performed using anti-HA agarose on lysates derived from 293T cells exogenously expressing Flag-tagged RNF41 and HA-tagged CACYBP (D), or Flag-tagged CACYBP and HA-tagged RNF41 (E). The asterisk indicates a nonspecific band. (F) An endogenous immunoprecipitation assay was performed using Huh7 cell extract and anti-CACYBP antibody. Rabbit normal anti-IgG was used as a control. Western blotting was performed with the indicated anti-CACYBP or anti-RNF41 antibody. (G-H) Immunoprecipitation and western blotting experiments were performed using anti-HA agarose on lysates derived from 293T cells exogenously expressing Flag-tagged CACYBP and the indicated HA-tagged RNF41 truncated mutants (G), or Flag-tagged RNF41 and the indicated HA-tagged CACYBP truncated mutants (H).

CACYBP is known to exert its function through binding various proteins 5. To find out the possible upstream or downstream regulators of CACYBP, 293T cells were used to express SFB-tagged CACYBP and after a two-step TAP of exogenous SFB-CACYBP, the immunoprecipitated complex was analyzed by silver staining (Figure 3A) and the mass spectrometry. Among the results, we identified a group of molecules that were reported to be associated with the ubiquitin regulation pathway, such as RNF41, UBB, UBC, STUB1, CULLIN1, and TRAF3IP1 (Table S2 and Figure S4). We then focused on the E3 ubiquitin ligase RNF41 since it had a relatively higher coverage rate and unique peptide number (Figure 3B and 3C). We first evaluated whether CACYBP was able to bind RNF41. In the immunoprecipitation assay, exogenously expressing Flag-tagged RNF41 or CACYBP could be eluted with HA-tagged CACYBP or RNF41, respectively (Figure 3D and 3E). Moreover, by using a specific antibody against CACYBP, we also demonstrated that RNF41 was efficiently precipitated with endogenous CACYBP in Huh7 cells (Figure 3F). A previous report stated that RNF41 recruits E2s *via* its N-terminal half (residues 1-133) and substrates *via* its C-terminal half (residues 134-317) 17. We thus constructed the HA-tagged RNF41 N-terminus or C-terminus truncated mutants accordingly. When HA-tagged RNF41-NT or RNF41-CT mutant was co-transfected with Flag-tagged CACYBP into 293T cells respectively, only the RNF41-CT mutant was able to precipitate with Flag-tagged CACYBP (Figure 3G), suggesting that CACYBP may be recruited by RNF41 as a substrate for ubiquitination. Similarly, we constructed HA-tagged CACYBP-NT and CACYBP-CT mutants according to Matsuzawa's research 4, in which they reported that CACYBP could bridge Siah-1 to Skp-1, using its N-terminal region to bind Siah-1 and its C-terminal region to interact with Skp-1. However, when these mutants were co-transfected with Flag-tagged RNF41 separately, neither CACYBP-NT nor CACYBP-CT mutant precipitated with full-length RNF41 (Figure 3H). Taken together, these results demonstrate that RNF41 selectively interacts with CACYBP through its C-terminal half.

### RNF41 promotes the degradation of CACYBP

**Figure 4:**
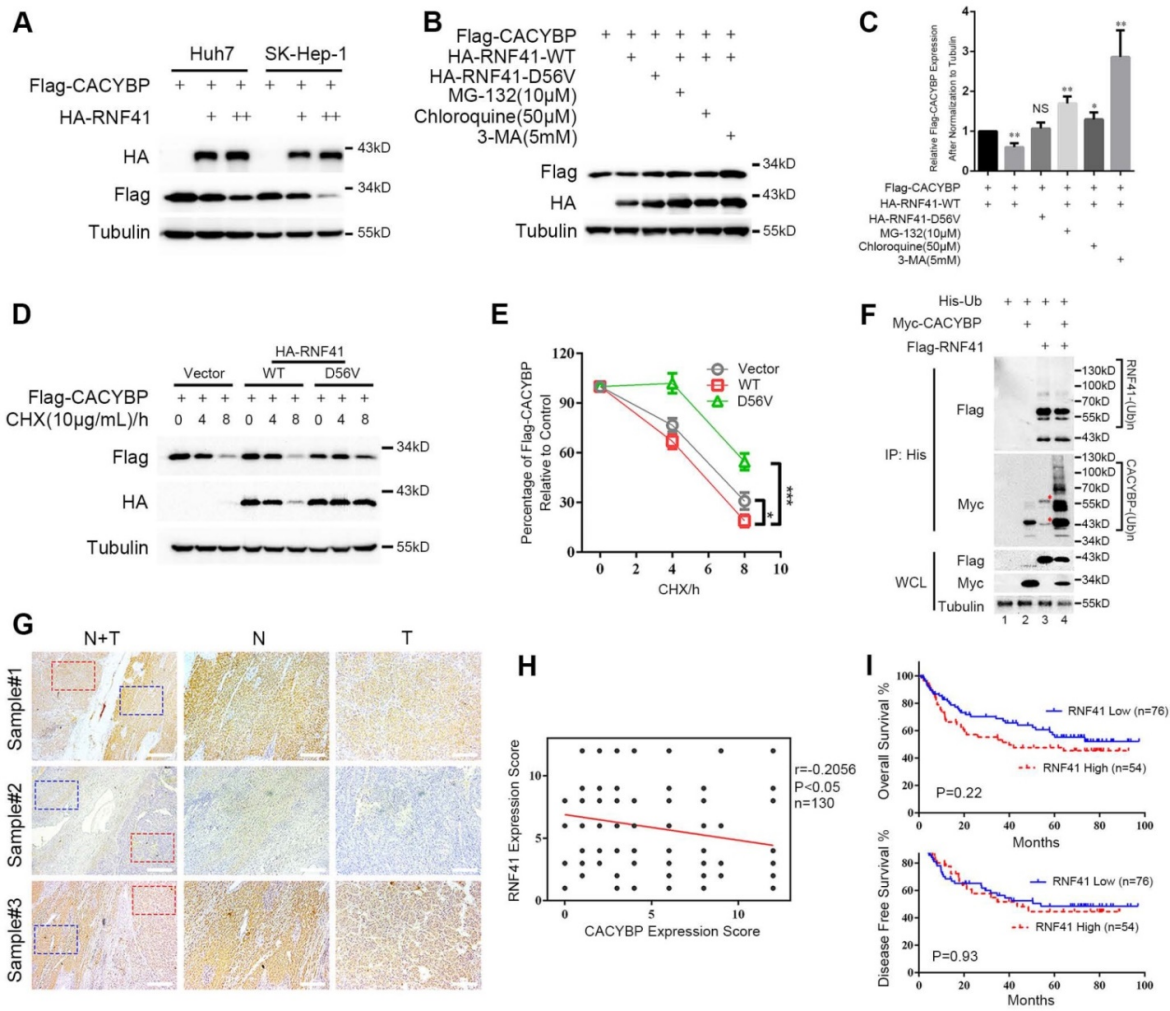
** RNF41 promotes the degradation of CACYBP.** (A) Expression level of Flag-tagged CACYBP was steadily reduced with increasing amounts of RNF41 in Huh7 and SK-Hep-1 cells. (B) RNF41 with E3 ligase activity reduced the accumulation of exogenously expressed CACYBP, but this did not occur in the presence of the proteasome inhibitor MG132 or the lysosome inhibitors chloroquine or 3-MA. 293T cells were transfected with the indicated plasmids and incubated for 24 h. The cells were then incubated in fresh medium with none, 10 μM MG-132, 50 μM chloroquine, or 5 mM 3-MA for an additional 12 h. The cell lysates were analyzed by immunoblotting using the indicated antibody. Tubulin was used as a loading control. (C) Quantification of the expression level of Flag-tagged CACYBP after normalization to Tubulin. (D) Protein turnover of exogenously expressing Flag-tagged CACYBP in the presence of RNF41 WT or D56V mutant over the course of 8 h following the addition of 10 μg/mL cycloheximide. Tubulin was used as a loading control. (E) Quantification of the percentage of Flag-tagged CACYBP in the presence of RNF41 WT or D56V mutant for each time point compared to 0 h in (D). (F) RNF41 stimulates the poly-ubiquitination of CACYBP* in vivo*. 293T cells were transfected with the indicated plasmids and incubated for 24 h. Cell lysates were subjected to immunoprecipitation with His affinity nickel beads. Immunoblotting analysis was conducted for the indicated proteins. The red asterisks in lane 3 indicate nonspecific bands. (G) Representative images of RNF41 expression from the same sample slices used for examining CACYBP expression by immunohistochemistry analysis. Scale bar for the left column: 400 μm; Scale bar for the middle and right columns: 100 μm. T: tumor; N: non-tumor. (H) Scatterplot of expression scores of CACYBP *vs* RNF41 with a regression line showing a negative correlation. (I) RNF41 expression showed no significant association with OS (upper panel) and DFS (lower panel) in the HCC cohort according to Kaplan-Meier analysis. RNF41 high: 76 samples; RNF41 low: 54 samples. *P < 0.05; ***P < 0.001.

Because RNF41 binds CACYBP, we then examined whether RNF41 functions as an E3 ligase and stimulates the degradation of CACYBP. We first found that Flag-tagged CACYBP was steadily reduced when co-transfected with increasing amounts of HA-tagged RNF41 in Huh7 and SK-Hep-1 cells (Figure 4A). Next, 293T cells were co-transfected with Flag-tagged CACYBP and HA-tagged RNF41 wild type (WT), E3 ligase-dead D56V mutant 17, or WT in the presence of proteasome inhibitor MG-132, or lysosome inhibitors chloroquine or 3-MA as indicated. The results showed that compared to the control, RNF41 WT caused a decrease in the expression of Flag-tagged CACYBP, while D56V mutant did not. Treatment of MG-132, chloroquine and 3-MA all obviously increased the expression of Flag-tagged CACYBP, suggesting that RNF41 promoted the degradation of CACYBP *via* both proteasome- and lysosome-dependent pathways (Figure 4B and 4C). Cycloheximide chase analysis also revealed that compared to the control, CACYBP was degraded faster when co-expressed with RNF41 WT, but much slower with D56V mutant (Figure 4D and 4E). To determine whether RNF41 causes ubiquitination of CACYBP, in 293T cells transfected with Myc-tagged CACYBP and ubiquitin, RNF41 WT co-transfection remarkably stimulated the formation of poly-ubiquitin conjugates of CACYBP, and RNF41 autoubiquitination showed little change with or without CACYBP co-expression (Figure 4F). Furthermore, we detected RNF41 expression in 130 HCC tissue slices within the same cohort we used for CACYBP examination by immunohistochemistry. In the same sample slices presented in Figure 1E, RNF41 expression was higher in the non-tumor tissues compared to the tumor tissues, forming an reciprocal pattern to CACYBP expression (Figure 4G).

A weak but statistically significant negative association was observed when performing correlation analysis using expression scores of RNF41 and CACYBP in HCC tumor tissues (Figure 4H). We also tried to identify whether RNF41 expression was related to the prognosis of HCC patients. However, by Kaplan-Meier analysis, RNF41 expression showed no significant association with the OS or DFS in our HCC cohort (Figure 4I). Taken together, these results demonstrate that RNF41 ubiquitinates CACYBP and promotes its degradation *via* proteasome and lysosome dependent pathways in HCC cells.

### RNF41-CACYBP axis regulates the cytoplasm-nucleus transit of P27^Kip1^ in HCC cells

**Figure 5:**
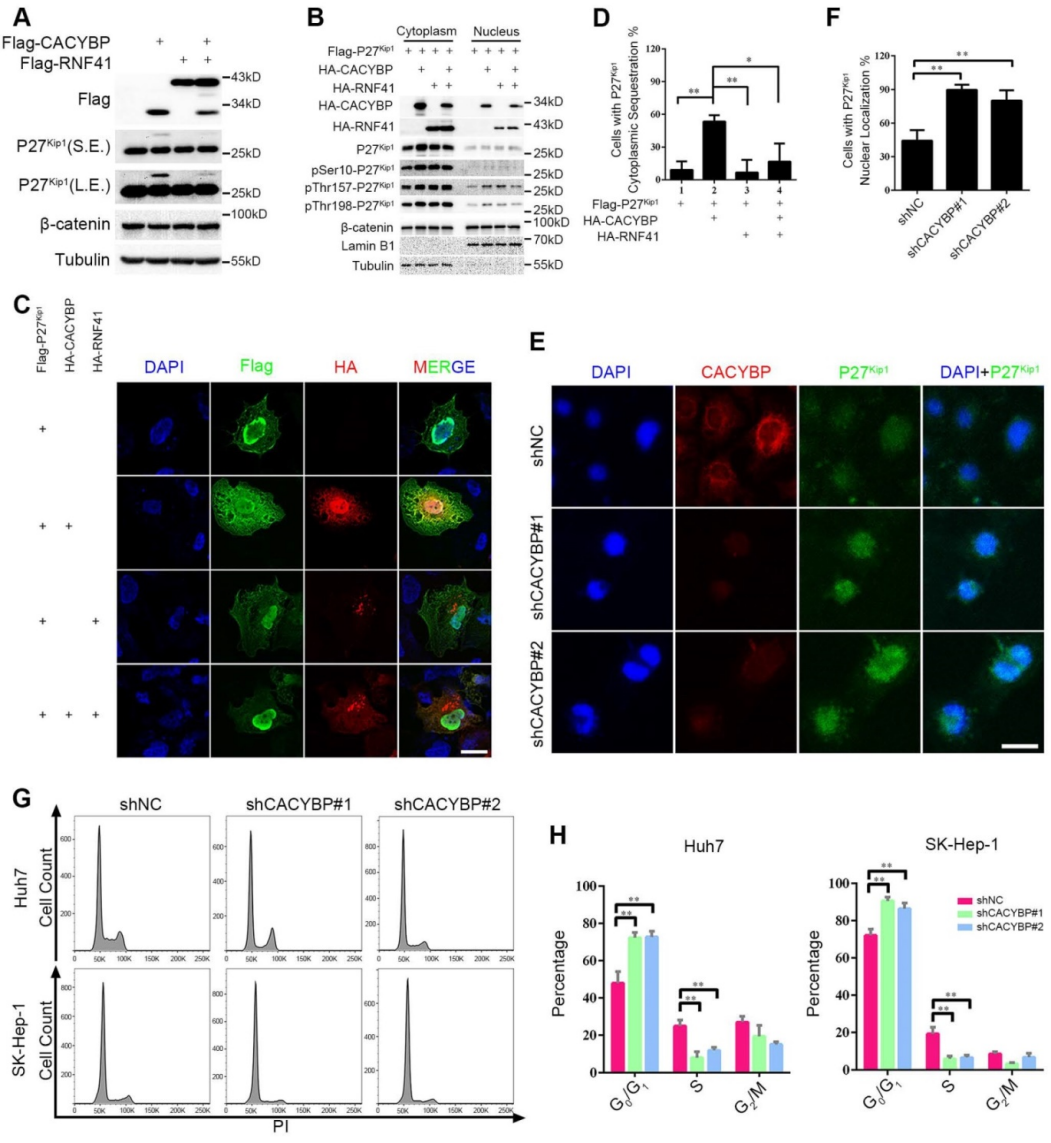
** RNF41-CACYBP axis regulates the cytoplasm-nucleus transit of P27^Kip1^ in HCC cells.** (A) CACYBP caused phosphorylation-like modification of P27^Kip1^, whereas RNF41 co-expression decreased it. Huh7 cells were transfected with the indicated plasmids and incubated for 24 h. Cell lysates were subjected to immunoblotting analysis with the indicated antibodies. β-catenin or tubulin was used as a negative control or loading control, respectively. S.E., short exposure; L.E., long exposure. (B) P27^Kip1^ level was increased in the cytoplasmic fraction upon CACYBP overexpression but decreased after RNF41 co-expression. Huh7 cells were transfected with the indicated plasmids and incubated for 24 h. Nuclear and cytoplasmic extracts were prepared by subcellular fractionation and subjected to immunoblotting analysis with the indicated antibodies. β-catenin was used as a negative control. (C) Immunofluorescence analysis of the cellular localization of P27^Kip1^ after overexpression of CACYBP and/or RNF41. Huh7 cells were transfected with the indicated plasmids and incubated for 24 h. After cell fixation, P27^Kip1^ was detected by anti-Flag immunostaining (green staining). CACYBP and RNF41 were detected by anti-HA immunostaining (red staining). Exogenous HA-tagged CACYBP showed a ubiquitous expression pattern in the nucleus and cytoplasm, and exogenous HA-tagged RNF41 showed a dotted expression pattern. Nuclear staining was performed using DAPI (blue staining). Scale bar: 10 μm. (D) Quantification of the percentage of cells with P27^Kip1^ cytoplasmic sequestration in (C). (E) Immunofluorescence analysis of the cellular localization of P27^Kip1^ in CACYBP-depleted Huh7 cells. Endogenous P27^Kip1^ and CACYBP were detected by anti-P27^Kip1^ (green staining) or anti-CACYBP (red staining) immunostaining, respectively. Nuclear was stained by DAPI (blue staining). Scale bar: 10 μm. (F) Quantification of the percentage of cells with predominantly nuclear localization of P27^Kip1^ in (E). (G) Representative images of the cell cycle distributions of control or CACYBP-depleted SK-Hep-1 and Huh7 cells. (H) Quantification of the percentage of cells in different cell cycle phases in (G). *P < 0.05; **P < 0.01.

To identify the signaling events influenced by CACYBP expression in HCC, we focused on two known CACYBP downstream factors, P27^Kip1^ and β-catenin. Total protein amounts of P27^Kip1^ and β-catenin were not altered by expressing CACYBP and/or RNF41 in Huh7 cells except that CACYBP overexpression caused an upwards shifted band of P27^Kip1^ not seen in the control and RNF41 only lanes, whereas RNF41 co-expression decreased the amount of this shifted band (Figure 5A). The band shift likely represents phosphorylation modifications of P27^Kip1^ whose cytoplasm-nucleus transit is mostly subjected to the regulation of phosphorylation status at residues Ser10, Thr157 or Thr198 31. We therefore analyzed the cellular distribution of P27^Kip1^ in the presence of CACYBP and/or RNF41 overexpression. Using the cytoplasmic and nuclear fractionation method, we observed that total P27^Kip1^ level was increased in the cytoplasmic fraction upon CACYBP overexpression but decreased to the control level after RNF41 co-expression. CACYBP or RNF41 overexpression alone or in combination had no influence on the cellular distribution of β-catenin (Figure 5B). Consistent with the previous reports that Ser10 phosphorylation is required for P27^Kip1^ nuclear export 32, and reports that Thr157 and Thr198 phosphorylation causes its cytoplasmic retention 33, CACYBP overexpression led to elevated levels of p-Ser10-, p-Thr157- and p-Thr198-P27^Kip1^ in the cytoplasm, and RNF41 co-expression reduced only the level of p-Ser10-P27^Kip1^ but not the levels of p-Thr157- and p-Thr198-P27^Kip1^ (Figure 5B). Immunofluorescence analysis also confirmed that CACYBP overexpression caused a significantly higher amount of cells with cytoplasmic sequestration of P27^Kip1^, and RNF41 co-expression attenuated this phenomenon (Figure 5C, 5D and Figure S5A). In addition, CACYBP-depleted cells by shCACYBP#1 and shCACYBP#2 treatments showed predominantly nuclear localization of P27^Kip1^ compared to the control cells (Figure 5E, 5F and Figure S5B). To determine if cell cycle was affected by CACYBP expression in HCC cells, flow cytometry analysis was performed and the data revealed that CACYBP knockdown SK-Hep-1 and Huh7 cells displayed a significantly higher percentage of G1 phase cells and a lower percentage of S phase cells (Figure 5G and 5H), suggesting that CACYBP depletion caused typical cell cycle arrest at the G1/S phase in HCC cells. Taken together, these results indicate that CACYBP induces the cytoplasmic retention of P27^Kip1^ and promotes cell cycle progression in HCC cells, but RNF41 works as a brake against CACYBP function.

### Ser10 phosphorylation of P27^Kip1^ mediates the effect of CACYBP on HCC cells

**Figure 6:**
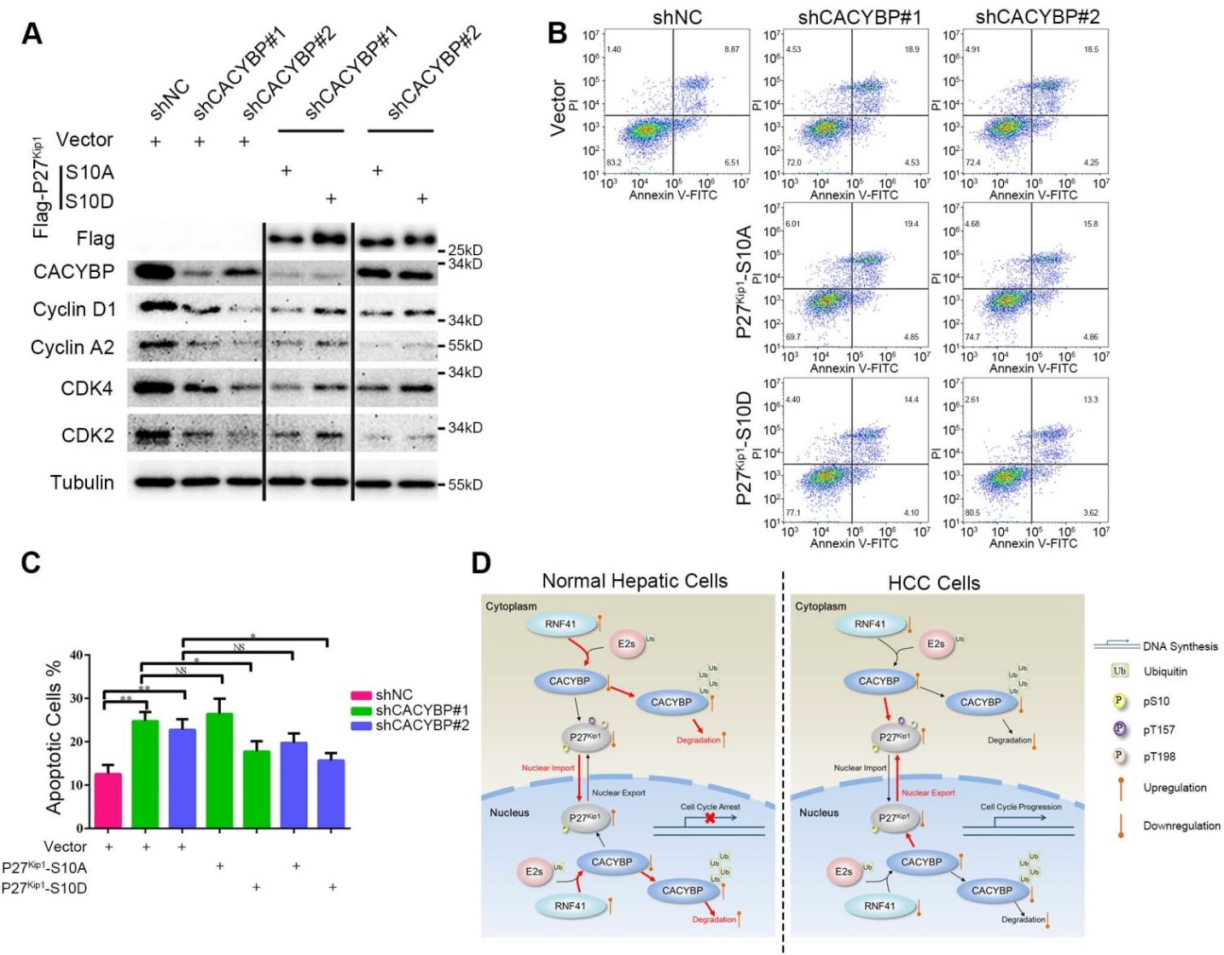
** Ser10 phosphorylation of P27^Kip1^ mediates the effect of CACYBP on HCC cells.** (A) Western blotting analysis of cell cycle proteins after reconstitution with P27^Kip1^ phosphomimetic mutants in CACYBP-depleted SK-Hep-1 cells. Cells were transfected with the indicated plasmids and incubated for 24 h. Cell lysates were subjected to immunoblotting analysis with the indicated antibodies. (B) P27^Kip1^-S10D overexpression rescued the apoptotic phenotype after CACYBP depletion in SK-Hep-1 cells. Cells were transfected with the indicated plasmids and incubated for 24 h. Detection of apoptosis was performed by concurrent staining with Annexin V-FITC and PI. (C) Quantification of the percentage of apoptotic cells in (B). (D) Schematic diagram of CACYBP-mediated HCC progression. *P < 0.05; **P < 0.01; NS, not significant.

To confirm whether Ser10 phosphorylation status of P27^Kip1^ was associated with CACYBP function in HCC cells, we constructed two P27^Kip1^ phosphomimetic mutants S10A and S10D, which preferentially adopt nuclear and cytoplasmic localization respectively 34, and reconstituted them in CACYBP-depleted cells. Compared to control cells, CACYBP-depleted cells showed reduced levels of proteins required for the G1/S phase transition, such as cyclin D1, cyclin A2, CDK2 and CDK4. The amount of these proteins was not affected by reconstitution with P27^Kip1^-S10A, whereas, reconstitution with P27^Kip1^-S10D partially increased the expression of these proteins in CACYBP-depleted cells (Figure 6A), indicating a recovery of cell cycle function. In addition, P27^Kip1^-S10A overexpression did not affect the apoptotic rate of CACYBP depleted cells. However, P27^Kip1^-S10D overexpression rescued the apoptotic features and decreased the percentage of dying cells after CACYBP depletion (Figure 6B and 6C). Taken together, these results demonstrate that, at least in part, phosphorylation of P27^Kip1^ at Ser10 mediates the effect of CACYBP on HCC cells.

## Discussion

In this study, we demonstrated that CACYBP expression is elevated in HCC and is associated with poor prognosis in patients. CACYBP depletion inhibits HCC cell proliferation and growth *in vitro* and *in vivo*. RNF41 has been identified as an upstream E3 ligase that ubiquitinates and promotes the degradation of CACYBP. Moreover, CACYBP exerts its tumor-promoting effect by releasing rapid cell cycle progression through promoting the Ser10 phosphorylation and subsequent cytoplasmic sequestration of P27^Kip1^, whereas RNF41 could act against CACYBP function in HCC. Therefore, our study provides the first novel view that CACYBP contributes to the development and progression of HCC, and may serve as a promising therapeutic and prognostic biomarker.

In contrast to the previous report 7 that CACYBP expression had no or obscure staining in 10 HCC tissues and was undetectable in normal liver tissues, our immunohistochemistry results showed that a relatively high amount of CACYBP expression was present in HCC tissues and a part of the adjacent non-tumor tissues. Two immortalized hepatic cell lines also showed positive expression. Because of the cancer-prone environment and property, CACYBP expression may have a lower but detectable level in tumor adjacent tissues and immortalized hepatic cell lines. In addition, due to the usage of different endogenous antibodies and samples, it is reasonable that discrepancies in CACYBP expression in liver tumors could be found by different studies. However, our results are supported by the result from the project of Human Protein Atlas 35 showing that high or medium CACYBP expression accounted for a large percentage of the examined HCC tissues (8 out of 12). Therefore, based on the immunohistochemistry results, our conclusions that CACYBP expression is elevated and associated with poor prognosis in HCC patients are authentic and reliable.

In fact, the interaction of CACYBP and RNF41 was predicted through a combination of mass spectrometry and quantitative high-throughput LUMIER assays by another group 36. However, we are the first to confirm their interaction by traditional immunoprecipitation method at both exogenous and endogenous levels. Just as it usually does, RNF41 recruits CACYBP through its substrate binding domain located at the C-terminus but not the N-terminal domain. However, deletion of either C-terminus (residues 81-229) or N-terminus (residues 1-73) abolished CACYBP's ability to immunoprecipitate with RNF41. It is likely that CACYBP uses a fragment containing both N-terminus and CS domains (residues 1-179) to bind RNF41 in a manner similar to the interaction with tubulin 37 or actin 38. It may also be true that CACYBP binds RNF41 requiring a proper structure through the cooperation of different domains, since CACYBP forms dimers in cells with an anti-parallel configuration mediated by the N-terminal domains to generate a range of architectures 39. Therefore, more studies are needed to confirm which segment of CACYBP protein mediates the interaction with RNF41.

When it came to the study of RNF41 expression in HCC tissues, our results showed that it was inversely correlated with CACYBP expression with a slightly significant P value. Consistency was found from the study by Shao *et al* 28 that RNF41 expression was reduced in HCC tumor tissues compared with adjacent healthy tissues. However, they also claimed that its expression corresponded to a higher survival rate of HCC patients, which was different from our finding that RNF41 expression had no obvious correlation with patient survival. Indeed, reduced RNF41 levels have been reported in and contribute to the progression of breast cancer 24, prostate cancer 25 and brain cancer 27. RNF41 may exert anti-tumor effects through downregulation of ERBB2/3, BRUCE or PCP component, thus reducing cell proliferation or motility in the above-stated cancers. Similarly, our results indicated that RNF41 may also act as a tumor suppressor by degrading CACYBP in HCC. Whether RNF41 performs a consistently inhibitory role in other human cancers awaits further investigation.

P27^Kip1^ inhibits cell cycle entry by targeting a broad spectrum of cyclin-dependent kinases and downregulation of the protein levels, particularly its nuclear expression level, is reported to be associated with both disease progression and poor prognosis in a wide range of cancers 40-42, suggesting that the regulation of subcellular localization is important for its function. Phosphorylation modifications predominantly mediate the regulation of P27^Kip1^'s cellular localization, among which Ser10 phosphorylation promotes P27^Kip1^ nuclear export *via* CRM1 protein 34, 43, and Thr157 or Thr198 phosphorylation, in a less common way, impairs P27^Kip1^ nuclear import under serum stimulation 44. As P27^Kip1^ is a known downstream target of both CACYBP and RNF41, a previous study has described that CACYBP aids in the degradation of cytoplasmic P27^Kip1^ by Siah1 45, and RNF41 reduces the level of cytoplasmic P27^Kip1^ *via* suppression of ERBB3-Akt signaling 46, thus causing effects on the migration and invasion abilities of glioma cells. Apart from increasing cellular migratory activity, cytoplasmic P27^Kip1^ has also been reported to suppress apoptosis in breast cancer 47 and enhance proliferation in trophoblast cells 48. Our study revealed that overexpression of CACYBP and/or RNF41 had no effect on the total amount of P27^Kip1^ in HCC cells, but CACYBP overexpression caused increased phosphorylation levels at Ser10, Thr157 and Thr198, and subsequent cytoplasmic retention of P27^Kip1^, through which CACYBP may promote HCC cell growth and proliferation. On the other hand, RNF41 co-expression reduces the phosphorylation level at Ser10, but not at the other two sites, resulting in partial nuclear recovery of P27^Kip1^ and likely acting as a brake against CACYBP's functions. A schematic model of HCC progression caused by CACYBP overexpression is shown in Figure 6D. In HCC cells, in the absence of enough RNF41, elevated CACYBP induces the phosphorylation of P27^Kip1^ at Ser10 and enhances its cytoplasmic retention, leading to rapid cell cycle progression and uncontrolled cell growth. However, in normal hepatic cells, RNF41 promotes the degradation of CACYBP *via* E3 ligase activity and counteracts its tumor-promoting effect. Therefore, we explore a novel mechanism for the actions of RNF41 and CACYBP on the regulation of P27^Kip1^ and demonstrate that phosphorylation regulation of P27^Kip1^ by RNF41-CACYBP axis is important for HCC progression.

In summary, we provide evidence that overexpression of CACYBP in HCC inhibits the cell cycle-restricted role of P27^Kip1^ and enhances tumor progression. The degree of activation of the RNF41/CACYBP/P27^Kip1^ axis has an impact on the survival probability of HCC patients *in vivo* and *in vitro* and may thus represent a promising molecular target.

## Materials and Methods

### Patient samples

We obtained a total of 136 paraffin-embedded HCC specimens for prognostic survival analysis from Sun Yat-sen University Cancer Center (Guangzhou, China), among which only 52 cases had both observable tumor tissues and adjacent non-tumor tissues under microscopy. To compare CACYBP expression between HCC tissues and adjacent non-tumor tissues, another 33 fresh HCC specimens were collected from the Third Affiliated Hospital of Sun Yat-sen University (Guangzhou, China). A surgical tumor resection was performed on each patient in the department of hepatobiliary surgery. Then tissues were cut into the proper size and fixed in 4% paraformaldehyde or stored in RNAlater solution (AM7020, Invitrogen) for immunohistochemistry analysis of CACYBP and RNF41 expression or quantification of CACYBP mRNA expression, respectively. The study was approved by the Institute Research Ethics Committee at the Sun Yat-sen University Cancer Center and the Third Affiliated Hospital of Sun Yat-sen University. Written informed consent was obtained from each patient. Relative experiments with these samples were performed in accordance with the relevant regulations.

### Reagents

Commercially available antibodies were as follows: CACYBP (11745-1-AP, Proteintech, 1:2000 for WB, 1:1000 for IF and 1:400 for IHC), Tubulin (66031-1-Ig, Proteintech, 1:5000 for WB), GAPDH (60004-1-Ig, Proteintech, 1:2000 for WB), Lamin B1 (66095-1-Ig, Proteintech, 1:1000 for WB), anti-Flag (F3165, Sigma, 1:3000 for WB and 1:1000 for IF), anti-HA tag (3724, Cell Signaling Technology, 1:1000 for WB and 1:500 for IF), anti-Myc tag (2276, Cell Signaling Technology, 1:1000 for WB), RNF41 (GTX115366, GeneTex, 1:1000 for WB and 1:200 for IHC), β-catenin (8480, Cell Signaling Technology, 1:1000 for WB), P27^Kip1^ (610241, BD Biosciences, 1:1000 for WB and 1:500 for IF), phosphor-Ser10-P27^Kip1^ (ab62364, Abcam, 1:1000 for WB), phosphor-Thr157-P27^Kip1^ (AF1555, R&D Systems, 1:1000 for WB), phosphor-Thr198-P27^Kip1^ (AF3994, R&D Systems, 1:1000 for WB), cyclin D1 (2978, Cell Signaling Technology, 1:1000 for WB), cyclin A2 (4656, Cell Signaling Technology, 1:1000 for WB), CDK2 (2546, Cell Signaling Technology, 1:1000 for WB), CDK4 (12790, Cell Signaling Technology, 1:1000 for WB). Small molecule inhibitors were as follows: MG-132 (474790, Calbiochem), chloroquine (S4157, Selleck), 3-methyladenine (3-MA, M9281, Sigma-Aldrich), cycloheximide (CHX, C7698, Sigma-Aldrich). All other chemical reagents were obtained from Sigma-Aldrich, unless otherwise indicated.

### Cell culture

293T cells, three HCC cell lines (SK-Hep-1, Huh7 and MHCC-LM3) and two immortalized hepatic cell lines (MIHA and LO2) were employed in this study and were cultured in Dulbecco's Modified Eagle's Medium (DMEM, Invitrogen) containing 10% fetal bovine serum (FBS, Gibco) at 37℃ and 5% CO_2_. All cell lines were obtained from the College of Life Sciences, Sun Yat-sen University. Cells were digested and passaged as previously described 49.

### Reverse transcription and quantitative PCR (qPCR)

Total RNA was isolated from tissue specimens using Trizol reagent (Invitrogen) according to the manufacturer's protocol. Total RNA (1 µg) was reverse transcribed into cDNA by the GoScript Reverse Transcription System (Promega). PCR was performed with Platinum SYBR Green qPCR SuperMix-UDG (Invitrogen) with a LightCycler 480 PCR platform (Roche). Specific primers were as follows: CACYBP forward, 5'-CTCCCATTACAACGGGCTATAC-3' and CACYBP reverse, 5'-GAACTGCCTTCCACAGAGATG-3'; GAPDH forward, 5'-GGAGCGAGATCCCTCCAAAAT-3' and GAPDH reverse, 5'-GGCTGTTGTCATACTTCTCATGG-3'.

### Plasmid construction and transfection

The cDNA encoding full-length human CACYBP, RNF41, P27^Kip1^ or ubiquitin were amplified by PCR from a cDNA library of 293T cells and subcloned into the pcDNA3.1(+) vector (Invitrogen). During the PCR, the sequences encoding Flag tag (GATTACAAGGATGACGACGATAAG), HA tag (TACCCATACGATGTTCCAGATTACGCT), Myc tag (GAACAGAAACTGATCTCTGAAGAAGACCTG) or 6×His tag (CATCATCACCATCACCAC) were added after the start codon to the 5' end of the CDS region of the indicated protein. The CACYBP N-terminus mutant (CACYBP-NT) was generated by deleting amino acids 81-229. The CACYBP C-terminus mutant (CACYBP-CT) was generated by deleting amino acids 1-73. The RNF41 N-terminus mutant (RNF41-NT) was generated by deleting amino acids 134-317. The RNF41 C-terminus mutant (RNF41-CT) was generated by deleting amino acids 1-133. PCR-based site-directed mutagenesis was used to obtain cDNA fragments encoding E3 ligase-dead RNF41 mutant (D56V) and P27^Kip1^ phosphomimetic mutants (S10A and S10D). S-protein-Flag-Streptavidin binding peptide (SFB) tag coding sequence was synthesized by Fulengen Company (Guangzhou, China) according to the previous report 50, ligated in-frame to CACYBP cDNA and subcloned into pcDNA3.1(+) vector. The Flag-CACYBP-DsRed lentiviral expression vector was generated by subcloning CACYBP cDNA into pLVX-DsRed-Monomer-N1 vector (Clontech). Vectors expressing short hairpin RNA (shRNA) against CACYBP were generated by ligating targeting sequences into pLKO.1 vector (Sigma-Aldrich). Correct constructs were all confirmed by DNA sequencing. Transient transfection was performed using Lipofectamine 2000 (Invitrogen) following the manufacturer's suggested procedures.

For establishment of stable cell lines, lentiviruses were produced by co-transfecting constructed plasmids and the packaging plasmids psPAX2 and pMD2.G (Addgene) into 293T cells using Lipofectamine 2000 for 48 h. Culture supernatants were collected, filtered, concentrated and used to infect targeted cells with the addition of 8 μg/mL polybrene (Sigma-Aldrich). After 48 h of infection, infected cells were selected by 2 µg/mL puromycin (Merck) for 2 weeks and successful establishment was confirmed by western blotting. The shRNA sequences targeting CACYBP mRNA were as follows: shCACYBP#1, 5'-GATATGAAGCGAACCATTAAT-3' and shCACYBP#2, 5'-AAGAGTTACTCCATGATTGTG-3'. The non-targeting shRNA sequence served as a negative control: shNC, 5'-CAACAAGATGAAGAGCACCAA-3'.

### Western blotting

Cells were lysed in NETN buffer (20 mM Tris-HCl at pH 8.0, 100 mM NaCl, 1 mM EDTA, 0.5% Nonidet P-40) containing protease and phosphatase inhibitor cocktail (Thermo Fisher Scientific). The lysate protein concentration was measured using the BCA protein assay kit (Pierce); after normalization to equal amounts, 10 μg of each protein sample was separated by SDS-PAGE, transferred to polyvinylidene fluoride (PVDF) membranes, blocked by 5% non-fatty milk (232100, BD Biosciences) diluted in 1×PBST (phosphate buffered saline supplemented with 0.5% Tween-20) and probed with the indicated primary antibodies. The blots were then incubated with species-specific HRP-conjugated secondary antibodies (W4011 for rabbit and W4021 for mouse originated primary antibodies, Promega, 1:5000), and the immunoreactive bands were visualized by enhanced chemiluminescence (ECL). The primary antibodies were diluted in Primary Antibody Dilution Buffer (P0023A, Beyotime) and the secondary antibodies were diluted in 1×PBST. Tubulin, GAPDH or Lamin B1 were used as loading controls. Quantification of band densitometry was measured with ImageJ software.

### Immunofluorescence

Cells were plated on chamber slides, and fixed with 4% paraformaldehyde at room temperature for 5 min. After fixation, cells were permeabilized with 0.1% Triton X-100 for 5 min. Then cells were blocked with 10% FBS for 20 min and incubated with indicated primary antibodies at 4℃ overnight. The fluorescence-visualized secondary antibody (Invitrogen) was added and incubated for 60 min. Nuclear staining was performed with 50 ng/mL DAPI (4',6-diamidino-2-phenylindole, D21490, Invitrogen) for 5 min at room temperature. Fluorescence signal was imaged using Zeiss LSM710 confocal microscope.

### Immunoprecipitation

Cells were washed twice with ice-cold 1×PBS (phosphate buffered saline) and lysed with NETN buffer containing protease and phosphatase inhibitor cocktail. Protein samples (500-1000 μg) were incubated with 1-4 μg of antibody overnight at 4℃ and then applied to pre-washed agarose beads, conjugated with either Protein A or Protein G for 2 h at 4℃. Agarose beads were then washed five times with NETN buffer and immunoprecipitates were collected by boiling beads in 50 μl of 1×SDS sample buffer for 15 min. Finally, the supernatant was subjected to SDS-PAGE and western blotting analysis.

### Ubiquitination assay

The in vivo ubiquitination assay was performed as described elsewhere 51. In brief, 293T cells were transiently transfected with indicated plasmids for 36 h. Transfected cells were harvested by denatured buffer (6 M guanidine-HCl, 0.1 M Na_2_HPO_4_/NaH_2_PO_4_, 10 mM imidazole). The cell extract was then incubated with nickel beads (Ni-NTA, R90101, Invitrogen) for 3 h, washed, and subjected to western blotting analysis. Mono- and poly-ubiquitinated CACYBP or RNF41 proteins migrated slower than non-ubiquitinated CACYBP or RNF41 proteins on SDS-PAGE, respectively.

### *In vivo* degradation assay

293T cells transiently transfected with the indicated plasmids were treated with 10 µg/mL of CHX for the indicated periods. Cells were lysed in NETN buffer and the cell extracts were analyzed by western blotting using the indicated antibodies.

### Subcellular fractionation

Nuclear and cytoplasmic extracts were prepared by NE-PER nuclear and cytoplasmic extraction reagents (Pierce) according to the supplier's protocol.

### Flow cytometry

For cell cycle analysis, cells were harvested in fresh medium. Samples were washed in PBS, and then fixed in ice-cold 70% ethanol at -20°C overnight. Fixed cells were washed by cold PBS and stained with FxCycle™ PI/RNase Staining Solution (F10797, Invitrogen) for 30 min at room temperature in the dark and analyzed by flow cytometry. For apoptosis analysis, cells were stained with FITC Annexin V Apoptosis Detection Kit I (556547, BD Biosciences) and evaluated by flow cytometry according to the manufacturer's protocol. Briefly, 1×10^6^ cells were washed twice with PBS and stained with 5 μL Annexin V-FITC and 5 μL PI in 1×binding buffer for 15 min at room temperature in the dark. Apoptotic cells were determined using a Beckman-Coulter Flow Cytometry FC500. Both early (Annexin V positive and PI negative) and late (Annexin V positive and PI positive) apoptotic cells were included when assessing cell death. Data were analyzed with FlowJo software version 10.

### Proliferation assay

Cell proliferation rate was determined using Cell Counting Kit-8 (CCK-8) assay (CK-04, Dojindo) according to the manufacturer's protocol. In brief, cells were seeded in 5 replicates in a 96-well plate at a density of 1,000 cells and cultured with 100 μL DMEM containing 10% FBS per well. At the indicated time point, 10 μL of the CCK-8 solution was added to each well, and the cells were incubated for another 4 h at 37°C. Viable cells were counted every two days by reading the absorbance at 450 nm with a plate reader (ELx800, BioTek).

### Colony formation assay

Cells were seeded into six-well plates at a density of 1,000 per well and incubated at 37℃ under an atmosphere of 5% CO_2_. Culture medium was changed every 2 days. Two weeks later, the cells were fixed with methanol, stained with 0.5% crystal violet (C6158, Sigma-Aldrich), and dried. Only clearly visible colonies (more than 50 cells) were counted under a light microscope. The test was repeated three times.

### *In vivo* animal study

All mice were handled according to the Guide for the Care and Use of Laboratory Animals. The procedures were approved by the Institutional Animal Care and Use Committee of Third Affiliated Hospital of Sun Yat-sen University. Female NSI mice 52 (kindly provided by Professor Peng Li from Guangzhou Institutes of Biomedicine and Health, Chinese Academy of Sciences) aged 6 weeks were used for tumor xenografts. All animals were housed in standard cages (5 animals per cage) under specific pathogen-free conditions. Rodent laboratory chow and tap water were provided ad libitum and maintained under controlled conditions at a temperature of 24 ± 1℃, a humidity of 50 ± 10%, and a 12:12 h light/dark cycle. Food and water were freely available throughout the experiments. The NSI mice were randomly divided into three groups (5 mice per group): shNC, shCACYBP#1 and shCACYBP#2 groups. Cells (5 × 10^6^ in 0.1 mL PBS) were injected subcutaneously into the hind flank of NSI mice. Mice were monitored every 12 h for the first 3 days after inoculation of tumor cells, and then daily thereafter. Tumor sizes were measured every 3 days. Mice were sacrificed 10 weeks post-injection *via* cervical dislocation, and tumors from every group were extracted and weighed. The perpendicular diameters of the tumors were measured using a caliper, and the tumor volume was calculated using the following formula: tumor volume = π/6 × large diameter × smaller diameter^2^. The following are general humane endpoints for animals that require euthanasia in this study: 20% decrease in normal body weight; the inability to reach food or water for more than 24 h; a tumor burden greater than 10% body weight or a tumor that exceeds 20 mm in any one dimension. All efforts were made to minimize animal suffering.

### Immunohistochemistry

Immunohistochemistry was performed as previously described 49. Briefly, all paraffin-embedded HCC samples were cut into 4-μm sections on a glass slide. Then, these slides were dried overnight at 37℃, deparaffinized in xylene twice for 10 min and rehydrated through graded alcohol five times for 5 min, and immersed in 3% hydrogen peroxide for 15 min to block endogenous peroxidase. The sections were boiled in an electric pressure cooker in ethylenediamine tetraacetic acid (EDTA) buffer (pH = 8.0) to retrieve antigen for 3 min. Next, the slides were incubated with 10% normal goat serum at room temperature for 30 min to reduce nonspecific reaction. Sections were then incubated overnight with primary antibodies against CACYBP or RNF41 at 4℃ and an anti-rabbit secondary antibody (K5007, Dako) at room temperature for 1 h. Signals were detected in freshly prepared DAB substrate solution (K5007, Dako) at room temperature for 5 min. Finally, the sections were counterstained with Mayer's hematoxylin, dehydrated, and mounted. Each section was evaluated by two independent pathologists blinded to the clinical status of patients and graded as described, according to the positive staining intensity scores (no staining, 0; weak staining, 1; moderate staining, 2; strong staining, 3) and the expression extent scores (< 25%, 1; 25-50%, 2; 50-75%, 3; > 75%, 4). A final expression score was defined as the intensity score multiplied by the extent score. All scores were subdivided into two categories according to a cutoff value of the ROC curve in the study cohort: CACYBP high expression (> 7.5) and low expression (< 7.5); RNF41 high expression (> 5.5) and low expression (< 5.5).

### Tandem affinity purification (TAP) of SFB-tagged protein complexes

293T cells were transfected with plasmids encoding SFB-tagged CACYBP. Successful expression of exogenous SFB-tagged CACYBP was confirmed by western blotting analysis. For affinity purification, transfected 293T cells were subjected to lysis in NETN buffer (with protease and phosphatase inhibitor cocktail) for 20 min at 4°C. Crude lysates were subjected to centrifugation at 14,000 rpm for 15 min at 4°C. Supernatants were incubated with Streptavidin Sepharose (GE Health) for 4 h at 4°C. The beads were washed three times with NETN buffer, and bound proteins were eluted with NETN buffer containing 2 mg/mL biotin (Sigma) at 4°C. The elute was incubated with S-protein agarose for 4 h (Novagen). The beads were washed three times with NETN buffer and subjected to SDS-PAGE. Protein bands were visualized by silver staining, excised and subjected to mass spectrometry (MS) analysis performed by WininnovateBio Company (Shenzhen, China).

### Liquid chromatography (LC)-MS/MS analysis and data interpretation

Resulting peptides were separated by reverse phase LC on an Eksigent nanoLC-Ultra™ 2D System (AB SCIEX). Data acquisition was performed with a Triple TOF 5600 System (AB SCIEX) fitted with a Nanospray III source (AB SCIEX) and a pulled quartz tip as the emitter (New Objectives). Based on combined MS and MS/MS spectra, proteins were successfully identified based on 95% or higher confidence interval of their scores in the MASCOT V2.3 search engine (Matrix Science Ltd.) with an overall false discovery rate for peptides of less than 1%. The identified MS/MS spectra were manually verified.

### Statistical analysis

The SPSS software version 20.0 and GraphPad Prism 6 software were used to perform statistical analyses. Correlation of the CACYBP staining intensity with clinicopathological characteristics was measured using Pearson's Chi-Square or Fisher's exact test. The Cox proportional hazards model and Kaplan-Meier analysis were employed for survival analysis. Spearman's correlation coefficient was employed for CACYBP and RNF41 correlation analysis. The significance of variances between groups was determined by t-test. Each experiment was performed three times in triplicate. Unless otherwise indicated, all error bars indicate standard deviation (SD). All statistical tests were two-sided, and P < 0.05 was considered statistically significant. P < 0.05, P < 0.01 and P < 0.001 were indicated by *, **, and *** in the figures, respectively.

## Supplementary Material

Supplementary figures and tables.

## Figures and Tables

**Table 1:** Association between CACYBP expression and clinicopathological parameters in 136 HCC specimens.

Variable	n	CACYBP low	CACYBP high	P
Age (years)				
≤ 45	63	33	30	0.430
> 45	73	38	35	
Gender				
Male	118	64	54	0.225
Female	18	7	11	
Tumor size (cm)				
≤ 5	45	26	19	0.246
> 5	87	41	46	
NA	4			
Capsular formation				
Negative	33	17	16	0.920
Positive	99	50	49	
NA	4			
Tumor thrombus				
Negative	123	65	58	0.646
Positive	13	6	7	
Tumor nodes				
Single	108	55	53	0.935
Multiple	24	12	12	
NA	4			
Ascites				
Negative	124	67	57	0.285
Positive	12	4	8	
Metastasis				
Negative	121	63	58	0.884
Positive	14	7	7	
NA	1			
TMN stage				
I-II	103	56	47	0.372
III-IV	33	15	18	
AFP (ng/mL)				
≤ 400	71	44	27	0.007*
> 400	60	23	37	
HBsAg				
Positive	114	58	56	0.769
Negative	17	8	9	
NA	5			
HBeAg				
Positive	123	62	61	0.982
Negative	8	4	4	
NA	5			
Cirrhosis				
Negative	29	19	10	0.072
Positive	103	48	55	
NA	4			
ALT (U)				
≤ 40	66	33	33	0.862
> 40	66	34	32	
NA	4			
AST (U)				
≤ 40	59	39	20	0.002*
> 40	73	28	45	
NA	4			
ALB (g/L)				
≤ 35	7	0	7	0.018*
> 35	125	67	58	
NA	4			
PT (s)				
≤ 14	100	51	49	0.952
> 14	31	16	15	
NA	5			
Survival				
Yes	74	48	26	0.001*
No	62	23	39	
Recurrence				
Yes	83	35	48	0.003*
No	53	36	17	

*P < 0.05 Abbreviations: AFP, alpha-fetoprotein; ALT, Alanine aminotransferase; ALB: albumin; AST, Aspartate transaminase; HBeAg, hepatitis B e antigen; HBsAg, hepatitis B surface antigen; NA, not available; PT, prothrombin time.

## Abbreviations

3-MA: 3-methyladenine
CACYBP: calcyclin-binding protein
CCK-8: Cell Counting Kit-8
CDK: cyclin-dependent kinase
CHX: cycloheximide
CS: CHORD and Sgt1
DAPI: 4',6-diamidino-2-phenylindole
DFS: disease free survival
DMEM: Dulbecco's Modified Eagle's Medium
EDTA: ethylenediamine tetraacetic acid
FBS: fetal bovine serum
HCC: hepatocellular carcinoma
IF: immunofluorescence
IHC: immunohistochemistry
IL-6R: interleukin-6 receptor
IP: immunoprecipitation
LC: liquid chromatography
LIFR: leukaemia inhibitory factor receptor
LR: leptin receptor
MS: mass spectrometry
Nrdp1: neuregulin receptor degradation protein-1
OS: overall survival
PBS: phosphate buffered saline
PBST: phosphate buffered saline supplemented with Tween-20
RING: Really Interesting New Gene
RNF41: RING finger protein 41
ROC: receiver operating characteristic
SFB: S-protein-Flag-Streptavidin binding peptide
SGS: Sgt1-specific
SIP: Siah1 interacting protein
TAP: tandem affinity purification
WB: western blotting
