# Supplementary material for: CACYBP Enhances Cytoplasmic Retention of P27Kip1 to Promote Hepatocellular Carcinoma Progression in the Absence of RNF41 Mediated Degradation
Source: Theranostics. 2019 Oct 22;9(26):8392–408. doi: 10.7150/thno.36838 (PMC6857042; doi:10.7150/thno.36838)
Supplement: Supplementary file 1 — Supplementary figures and tables. [file thnov09p8392s1.pdf]

Table S1. Univariate and multivariate analysis of overall survival in 136 HCC specimens

	Univariate analysis			Multivariate analysis		
	Hazard ratio	95% CI	P	Hazard ratio	95% CI	P
Age (years)	1.263	0.692-2.307	0.447			
Gender	1.479	0.562-3.890	0.428			
Tumor size (cm)	3.418	1.332-7.439	0.009*	2.813	1.413-5.598	0.003*
Capsular formation	0.768	0.339-1.742	0.528			
Tumor thrombus	3.458	1.211-9.874	0.020*	3.056	1.370-6.818	0.006*
Tumor nodes	2.594	0.915-7.356	0.073			
Ascites	0.448	0.124-1.610	0.218			
Metastasis	3.692	1.005-13.562	0.049*	2.889	1.376-6.066	0.005*
TMN stage	0.981	0.216-4.461	0.980			
AFP (ng/mL)	0.943	0.474-1.875	0.867			
HBsAg	1.055	0.384-2.903	0.917			
HBeAg	4.850	1.493-15.756	0.009*	3.181	1.187-8.523	0.021*
Cirrhosis	2.098	0.787-5.597	0.139			
ALT (U)	1.297	0.666-2.527	0.444			

AST (U)	1.273	0.614-2.638	0.516			
ALB (g/L)	0.225	0.047-1.067	0.060			
PT (s)	2.218	1.088-4.523	0.028*	2.133	1.124-4.046	0.020*
CACYBP expression	2.596	1.306-5.160	0.006*	3.246	1.794-5.874	<0.001*

*P < 0.05

Abbreviations: AFP, alpha-fetoprotein; ALT, Alanine aminotransferase; ALB: albumin; AST, Aspartate transaminase; HBeAg, hepatitis B e antigen; HBsAg, hepatitis B surface antigen; PT, prothrombin time.

Table S2. Summary of ubiquitination related proteins co-immunoprecipitating with SFB-tagged CACYBP from 293T cells, identified by MALDI-TOF.

Gene Name	Coverage%	Unique Peptide#	Description
CACYBP	89	66	-
RNF41	16	6	E3 ligase
UBB	19	5	Polyubiquitin-B
UBC	6	5	Polyubiquitin-C
STUB1	13	4	E3 ligase
CULLIN1	3	1	Component of E3 ligase complex
TRAF3IP1	2	1	E3 ligase interating protein

Figure S1

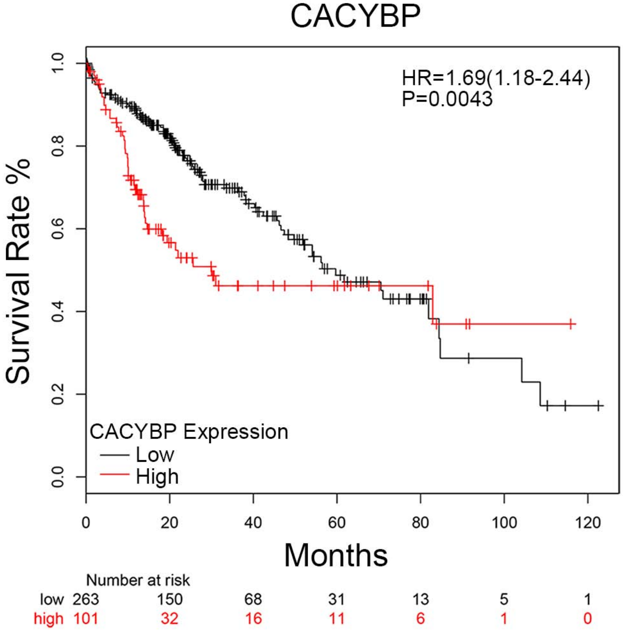
CACYBP
HR=1.69(1.18-2.44)
P=0.0043
Survival Rate %
1.0
0.8
0.6
0.4
0.2
0.0
CACYBP Expression
Low
High
0
20
40
60
80
100
120
Months
Number at risk
low 263 150 68 31 13 5 1
high 101 32 16 11 6 1 0

Figure S2

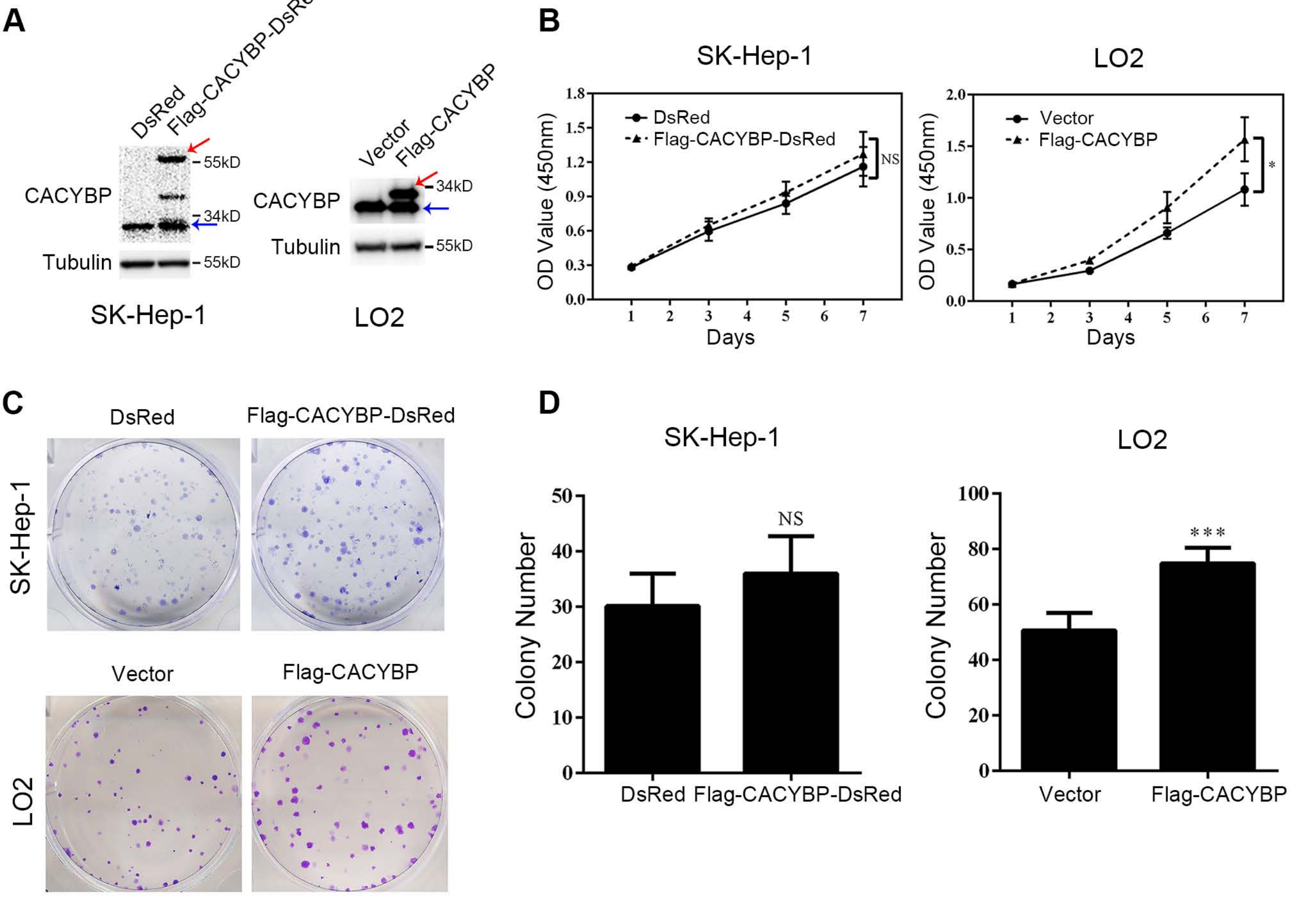
A
DsRed
Flag-CACYBP-DsRed
CACYBP
55kD
34kD
Tubulin
55kD
SK-Hep-1
Vector
Flag-CACYBP
CACYBP
34kD
Tubulin
55kD
LO2
B
SK-Hep-1
OD Value (450nm)
DsRed
Flag-CACYBP-DsRed
NS
Days
LO2
Vector
Flag-CACYBP
*
C
DsRed
Flag-CACYBP-DsRed
SK-Hep-1
Vector
Flag-CACYBP
LO2
D
SK-Hep-1
Colony Number
NS
DsRed
Flag-CACYBP-DsRed
LO2
***
Vector
Flag-CACYBP

Figure S3

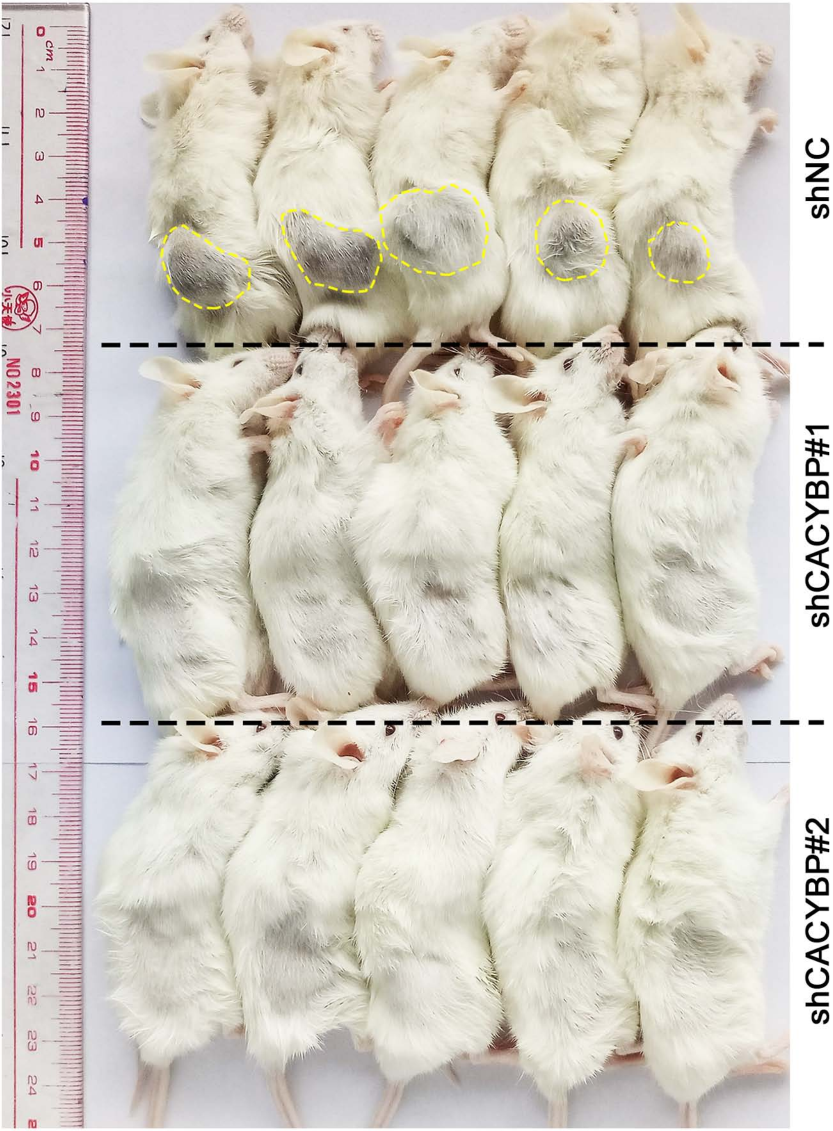

Figure S4

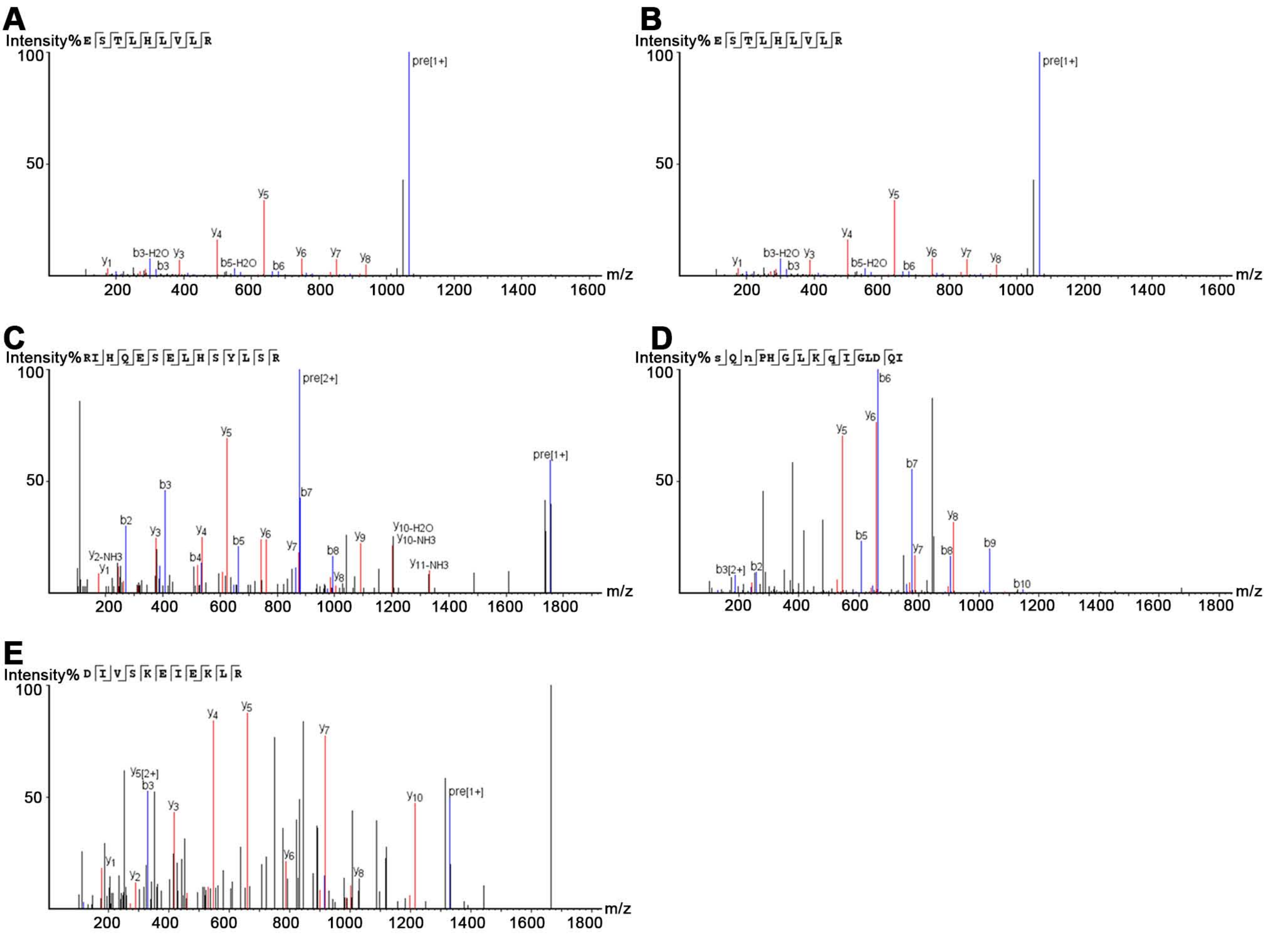
A
Intensity% E S T L H L V L R
pre[1+]
y5
y4
y1
b3-H2O
y3
b3
b5-H2O
b6
y6
y7
y8
m/z
B
Intensity% E S T L H L V L R
pre[1+]
y5
y4
y1
b3-H2O
y3
b3
b5-H2O
b6
y6
y7
y8
m/z
C
Intensity% R I H Q E S E L H S Y L S R
pre[2+]
y5
b3
b7
pre[1+]
b2
y3
y4
b5
y6
y7
b8
y8
y9
y10-H2O
y10-NH3
y11-NH3
y2-NH3
y1
b4
m/z
D
Intensity% s Q n P H G L K q I G L D Q I
b6
y6
y5
b7
y8
b5
b9
b8
y7
b3[2+]
b2
b10
m/z
E
Intensity% D I V S K E I E K L R
y4
y5
y7
y5[2+]
b3
y3
y10
pre[1+]
y1
y2
y6
y8
m/z

Figure S5

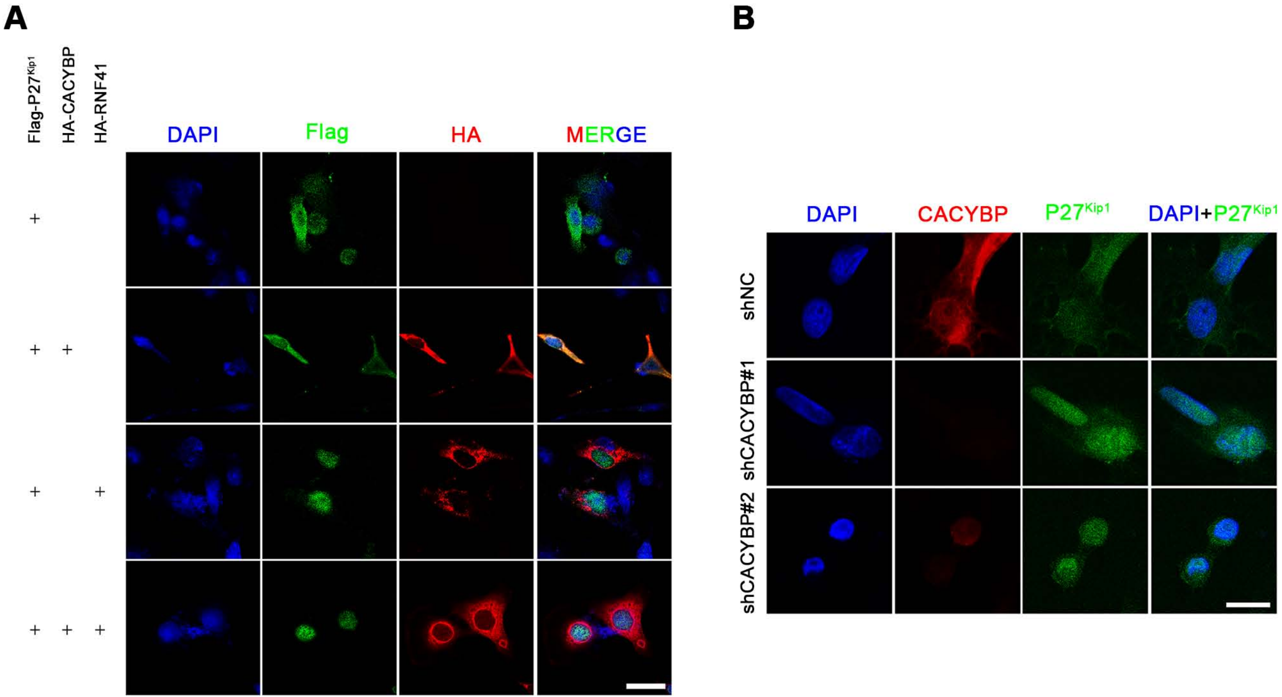
A
Flag-P27Kip1
HA-CACYBP
HA-RNF41
DAPI
Flag
HA
MERGE
+
+ +
+ +
+ + +
B
DAPI
CACYBP
P27Kip1
DAPI+P27Kip1
shNC
shCACYBP#1
shCACYBP#2
